# Genetically Modified T-Cell-Based Adoptive Immunotherapy in Hematological Malignancies

**DOI:** 10.1155/2017/5210459

**Published:** 2017-01-02

**Authors:** Baixin Ye, Creed M. Stary, Qingping Gao, Qiongyu Wang, Zhi Zeng, Zhihong Jian, Lijuan Gu, Xiaoxing Xiong

**Affiliations:** ^1^Department of Hematology, Renmin Hospital of Wuhan University, Wuhan, Hubei 430060, China; ^2^Department of Anesthesiology, Perioperative and Pain Medicine, Stanford University School of Medicine, Stanford, CA 94305-5117, USA; ^3^Department of Pathology, Renmin Hospital of Wuhan University, Wuhan, Hubei 430060, China; ^4^Department of Neurosurgery, Renmin Hospital of Wuhan University, Wuhan, Hubei 430060, China; ^5^Central Laboratory, Renmin Hospital of Wuhan University, Wuhan, Hubei 430060, China

## Abstract

A significant proportion of hematological malignancies remain limited in treatment options. Immune system modulation serves as a promising therapeutic approach to eliminate malignant cells. Cytotoxic T lymphocytes (CTLs) play a central role in antitumor immunity; unfortunately, nonspecific approaches for targeted recognition of tumor cells by CTLs to mediate tumor immune evasion in hematological malignancies imply multiple mechanisms, which may or may not be clinically relevant. Recently, genetically modified T-cell-based adoptive immunotherapy approaches, including chimeric antigen receptor (CAR) T-cell therapy and engineered T-cell receptor (TCR) T-cell therapy, promise to overcome immune evasion by redirecting the specificity of CTLs to tumor cells. In clinic trials, CAR-T-cell- and TCR-T-cell-based adoptive immunotherapy have produced encouraging clinical outcomes, thereby demonstrating their therapeutic potential in mitigating tumor development. The purpose of the present review is to (1) provide a detailed overview of the multiple mechanisms for immune evasion related with T-cell-based therapies; (2) provide a current summary of the applications of CAR-T-cell- as well as neoantigen-specific TCR-T-cell-based adoptive immunotherapy and routes taken to overcome immune evasion; and (3) evaluate alternative approaches targeting immune evasion* via* optimization of CAR-T and TCR-T-cell immunotherapies.

## 1. Introduction

A significant proportion of hematological malignancies remain limited in treatment options. Combinational therapeutics, such as chemotherapy in conjunction with targeted therapy by small molecules or monoclonal antibodies and/or hematological stem cell transplantation (HSCT), has led to a durable remission or even cure in some types of hematological malignancies [1]. While HSCT is currently considered to be the front-line option for treating most hematological malignancies, it can be accompanied by serious complications [1, 2]. Interestingly, graft-versus-leukemia response (GVL) in HSCT was reported to contribute to effective antitumor treatment [2, 3]. This observation provides compelling evidence that immune cells from the donor can significantly eliminate the malignant host cells in leukemia, lymphoma, and multiple myeloma. Therefore, modulating the immune system may be a potential therapeutic approach to combat hematological malignancies.

Cytotoxic T lymphocytes (CTLs) are an important subset of effector T-cells that act to mediate antitumor immunity by inducing cytolysis or apoptosis of malignant cells in a human leukocyte antigen- (HLA-) dependent manner. Unfortunately, hematological malignant cells can utilize multiple pathways to evade CTL-mediated immunity and evolve resistance to currently available combinational therapies, resulting in relapse or failure of treatment [1]. This immune evasion of hematological malignant cells can include impaired tumor antigen processing and presentation by tumor cells, dysfunction of antigen presenting cells (APCs), and defective costimulation and/or coinhibitory T-cell mediated pathways related to immune checkpoint blockade. In addition, expansion of suppressive immune cells, tumor altered metabolism, the production of regulatory soluble factors in tumor microenvironment, and downregulation of tumor cell surface antigens also facilitate immune escape from the CTL-mediated response [1, 2]. Overcoming tumor immune evasion may be a critical event in the successful treatment of specific hematological cancers. Therefore, understanding the detailed mechanisms of immune evasion is a necessary step in the development of novel immunotherapy approaches for these malignancies.

In solid tumors such as melanoma, tumor-infiltrating lymphocytes isolated from tumor tissues subjected to ex vivo expansion and subsequent transfusion back to the patient produced a partial antitumor effect [4, 5]. Despite similar success of allogeneic HSCT in treating or curing a majority of hematological malignancies, both allogeneic HSCT and adoptive transfer of tumor-infiltrating lymphocytes can lead to fatal complications or failure of treatment. This dilemma has prompted cancer immunologists to search for additional approaches to engineer CTLs to recognize and kill tumor cells specifically by counteracting tumor immune evasion. Currently, the genetically modified T-cell-based adoptive immunotherapies, including primarily engineered chimeric antigen receptor (CAR) gene-transduced T-cells (CAR-T) and T-cell receptor (TCR) gene-transduced T-cells (TCR-T), headlined advancements in clinical cancer therapy [6–8]. CAR is a fusion protein composed of an antibody derived extracellular single-chain variable fragment (scFv) with an antigen recognition moiety and an intracellular T-cell activation domain. T-cells with CAR expression can bind to the specific antigen and kill the tumor cells in an HLA-independent manner. Several clinic trials have demonstrated that CAR-T-cell-based adoptive immunotherapy produces a long-term remission in hematological malignancies that exceeds current standard combination therapies [7, 8].

Theoretically, CAR recognition is limited to the surface antigens in the context of HLA molecules. In contrast, engineered TCR gene-transduced T-cells can recognize intracellular proteins, which are processed and presented by antigen presenting cells (APCs) or tumor cells, in an HLA-dependent manner. Several lines of evidence suggest that hematological malignancies acquire tumor-associated mutations [9], some of which can generate* neoantigens* that can influence the antitumor response and serve as novel targets for adoptive immunotherapy [10, 11]. Neoantigen-specific CTLs are considered to work to kill tumor cells* via* presentation of neoantigen derived peptides in an HLA-dependent manner. Unfortunately, neoantigen-specific CTLs cannot be activated in the tumor altered microenvironment. Instead, engineered T-cells with expression of neoantigen-specific TCR can be expanded ex vivo and transfused to the patient, resulting in a specific TCR-T-cell-based immunity to eliminate the malignant cells [12]. Thus, the current advancement in genetically modified T-cell-based immunotherapy is a more specific approach to treat or cure hematological malignancies.

CAR-T and TCR-T-cell-based immunotherapies, which can interfere with a part of pathways responsible for immune evasion, may likely have limitations in their side effect profile [7, 8, 13]. Therefore, combining adoptive transfer of CAR-T or TCR-T-cells with other optimal measures such as chemotherapy, immune checkpoint blockade inhibition, and/or cytokine therapy, may provide a synergistic effect by simultaneously interfering with multiple pathways of immune evasion [14]. In addition, the rapid advancement of genome editing and gene transfer technology may also provide a promising platform for optimizing CAR-T or TCR-T-cell-based immunotherapeutics to achieve immune enhancement by altering gene expression in order to optimize immune response [14].

In order to summarize current findings in the application of genetically modified T-cell-based adoptive immunotherapies for hematological malignancies, we will first provide an overview of the current understanding of the multiple mechanisms for immune evasion by malignancies to avoid recognition by CTLs. Then, we will provide a detailed review on the application of CAR-T therapy and neoantigen-specific TCR-T-cell adoptive immunotherapeutics in overcoming immune evasion. Finally, we will evaluate measures targeting other pathways for immune evasion used to optimize the CAR-T or TCR-T-cell-based immunotherapy.

## 2. Tumor Immune Evasion in Hematological Malignancies

According to the tumor-immunoediting theory [15–17], the loss of equilibrium between tumor cell generation and immunity-mediated elimination results in tumor development secondary to immune evasion. Thus, understanding mechanistic details of immune evasion is necessary for the development of effective antitumor treatment. Distinguishing the self- or non-self-antigens is the basic characteristic of immune system [15]. In infectious diseases, exogenous antigens derived from pathogens can be engulfed and presented by APCs or the infected cells, thereby eliciting the specific CTLs to recognize and target the exogenous antigen in an HLA-restricted manner, resulting in death of the pathogens or their infected cells [18, 19]. Similarly, in hematological malignancies, targeted recognition on tumor cells by CTLs is the central step necessary for effective T-cell-mediated immunity [1]. Thus, impairing targeted recognition of CTLs on tumor cells is an important strategy for tumor immune evasion [1]. Immune evasion mechanisms include but are not limited to defective costimulation, immune checkpoint blockade, increased suppressive immune cells, tumor altered metabolism, regulated soluble factors, and impaired apoptosis-related pathways that are not directly related with targeted recognition of CTLs on tumor cells [1, 2]. Current advancements in strategies targeting tumor immune evasion include targeted recognition of CTLs on tumor cells and pathways independent of CTL specificity.

### 2.1. Strategies of Tumor Evasion That Are Closely Related with Targeted Recognition of Tumor Cells by CTLs

**Figure 1:**
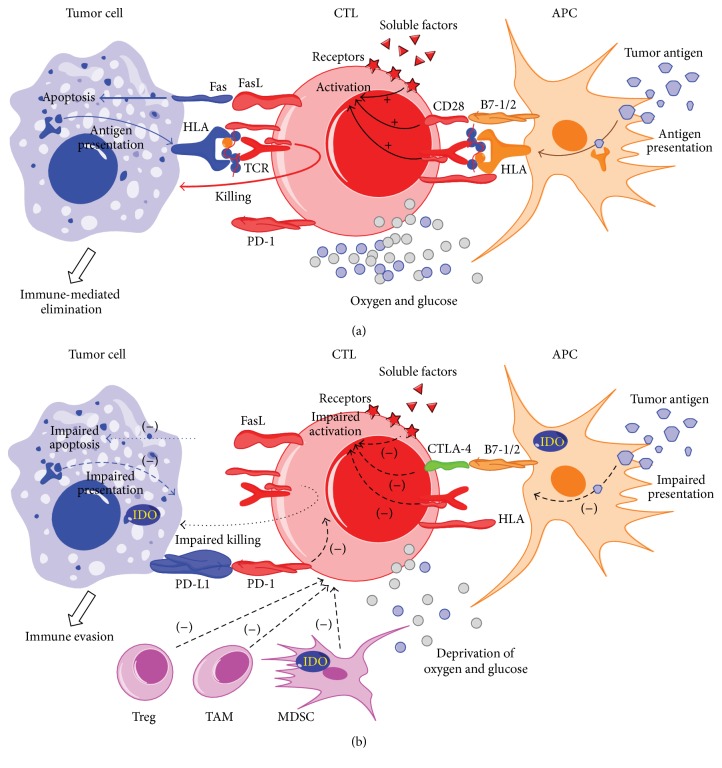
Immune-mediated elimination by cytotoxic T lymphocytes (CTLs) and tumor immune evasion strategies that are dependent on or independent of targeted recognition of CTLs on tumor cells in hematological malignancies. (a) Antigen presenting cells (APCs) uptake and present tumor antigens on the cell surface in an HLA-dependent manner, providing costimulatory signals (e.g., B7-1/2) for priming the T-cell response. Upon activation by APCs, CTLs can recognize the tumor cells with the presentation of tumor antigens in the context of proper metabolism (e.g., sufficient oxygen and glycose). Subsequently, CTLs kill tumor cells by releasing perforin and granzyme B or by expressing Fas ligand (FasL) on the surface, inducing cytolysis or apoptosis. (b) CTL-mediated immunity can be suppressed by targeted recognition-dependent and targeted recognition-independent mechanisms, leading to immune evasion in hematological malignancies. Strategies including dysfunctional APCs, defective costimulation, and impaired antigen presentation represent targeted recognition dependent immune evasion. In contrast, strategies including immune checkpoint pathways (e.g., CTLA-4 or PD-1/PD-1L), suppressive immune cells (e.g., Treg cell, tumor-associated macrophage TAM, myeloid-derived suppressor cell, and MDSC), tumor altered metabolism (IDO upregulation, oxygen, and glycose deprivation), and regulatory soluble factors (e.g., decreased IL-12) represent approaches independent of targeted recognition of CTLs on tumor cells.

In cellular immunity, APCs (including dendritic cells, macrophages, and subsets of B cells), phagocytose and present tumor antigens on the cell surface in an HLA-dependent manner, providing costimulatory signals for priming the T-cell response [1, 20]. Upon activation by APCs, CTLs can recognize tumor cells* via* HLA-dependent presentation of tumor antigens on the cell surface, resulting in CTL-mediated cell lysis or apoptosis (Figure 1(a)). In hematological malignancies, this process can be impaired, contributing to the loss of recognition of CTLs to malignant cells [1, 21–24].

The impaired targeted recognition of tumor cells by CTLs is primarily attributed to three mechanisms. First, dysfunctional APCs are usually insufficient for independent presentation of tumor antigens and fail to provide costimulation for priming tumor-specific CTLs [1]. It was reported that dendritic cells can promote antitumor immunity* via* uptake and presentation of altered self-antigens or neoantigens from malignant cells [25–27]. However, dendritic cells of patients with hematological malignancies can be decreased in quantity and quality by tumor cells or other components of tumor microenvironment [21, 22]. For example, tumor progression-related soluble factors, including cyclooxygenase-2 (COX-2), prostaglandin E_2_ (PGE_2_), transforming growth factor-*β* (TGF-*β*), and vascular endothelial growth factor (VEGF), can deregulate dendritic cell functions to impair the presentation of tumor antigens, interfering with activation of tumor-specific CTLs [28–30]. This results in dysfunction of APCs that indirectly impedes activation of tumor-specific CTLs, inhibiting T-cell-mediated elimination by interfering with targeted recognition of CTLs on tumor cells.

A second mechanism is described by defective antigen presentation of tumor cells in an HLA-dependent manner that contributes to the inability of CTLs to recognize malignant cells. Upon priming of APCs, the TCR of activated tumor-specific CTLs can recognize peptides derived from tumor antigens in the context of HLA molecules, leading to targeted killing of tumor cells. However, in hematological malignancies, it has been described that the expression of HLA on the surface of tumor cells was downregulated as a result of mutations or deletions in the* HLA* loci [1]. Given that B cells can present their own idiotypes in an HLA-dependent manner, it has been reported that a structural loss of HLA class I and II expression or mutations in* HLA classes I and II* loci facilitate the immune evasion of B-cell lymphoma cells [31]. Alternatively, mutations and deletions in the *β*2-microglobulin gene have been observed in Hodgkin lymphoma [32, 33]. Additionally, downregulation of genes associated with antigen presentation machinery has been described in lymphoma [1, 34].

A third strategy to escape from targeted recognition of CTLs can be described by the low rates of mutational recognition in hematological malignancies. Genome instability is generally a hallmark of tumor cells and can lead to somatic mutations that are entirely absent from the normal human genome across the whole genome-wide sequence [35]. In contrast with other tumors such as melanoma and lung cancers, hematological malignancies are 10–20 times lower in the frequency of mutations [10]. For example, multiple myeloma contains ~3000 somatic mutations, while acute myeloid leukemia, acute lymphocytic leukemia, and chronic lymphocytic leukemia each contain ~1500–2000 mutations. The reduced mutational load in hematological malignancies likely relates to the inactive T-cell response in the context of tumor progression. It was reported that only 0.3% to 1.3% of mutated peptides induced a CD8^+^ T-cell response and only 0.5% of mutated peptides elicited a CD4^+^ T-cell response [14]. Neoantigens, which are derived from this small part of somatic mutations, can elicit effective CTL response and likely play a key role in controlling tumor development [11, 15]. Mutation-derived neoantigens can be divided into two classes [15]: type *I* neoantigens can alter the amino acids in regions that make contact with the TCR normally without changing the anchor residues in relation to HLA molecules. These mutations do not change the binding affinity of the peptides to HLA molecules but may make the peptides immunogenic. In contrast, type* II* neoantigens are created from the mutations that can generate a new anchor residue, promoting the binding of the mutated peptide onto HLA complexes. Upon presentation by tumor cells, both types of neoantigens can be recognized by specific T-cells, followed by CTL-mediated killing of tumor cells. However, subdominant neoantigens that exist in hematologic malignancies cannot be efficiently presented, resulting in tumor immune evasion [14]. Attempts in inducing effective antitumor immunity requires high-avidity TCRs [14]. Additionally, neoantigen heterogeneity plays an important role in determining antitumor activity. It was recently reported that high degrees of neoantigen intratumor heterogeneity can produce a poor prognosis in non-small-cell lung cancer [11, 36]. Mutational load was also shown to positively correlate with antitumor immunity [15]. The improvement of mutational load has been considered responsible for an observed increase in cytotoxic therapy-induced subclonal neoantigens and improved outcomes in certain poor responders [11]. Neoantigen presentation might be also a determinant factor for influencing tumor evasion, although the exact details of this mechanisms remain to be determined [14].

### 2.2. Alternative Strategies of Immune Evasion

Alternative mechanisms of immune evasion independent of targeted recognition of tumor cells by CTLs (Figure 1(b)) include immune checkpoint pathways, regulatory soluble factors, suppressive immune cells and tumor altered metabolism, and factors promoting escape from immunity-mediated surveillance [1, 2]. Immune checkpoints, which refer to a number of inhibitory pathways, are critical for maintaining self-tolerance and modulating the immune response [20]. It was previously reported that tumor cells in hematological malignancies, such as multiple myeloma (MM) [37–39], non-Hodgkin lymphoma (NHL) [40], classic Hodgkin lymphoma (HL) [41], and myelodysplastic syndrome (MDS) [42, 43], can escape from the host immune system through immune checkpoints pathways, such as cytotoxic T-lymphocyte associated protein-4 (CTLA-4) and programed-death 1 (PD-1) pathways. Also, suppressive immune cells, including regulatory T-cells (Treg), tumor-associated macrophage (TAM), and myeloid-derived suppressor cells (MDSC), can form an inhibitory microenvironment surrounding the tumor cells [1, 44]. These cells can inhibit the response of leukemia-specific CTLs to the malignant cells by secreting soluble factors including inhibitory cytokines, such as interleukin-4 (IL-4), IL-10, and transforming growth factor *β* (TGF-*β*), as well as chemokines CCL22, CCL17, and CCL5 [45, 46]. Additionally, tumor altered metabolism can shape antitumor immunity [47]. For example, in tumor genesis, the derivation of glucose and amino acids caused by tumor growth can impair the proliferation and effector functions of T-cells, thereby promoting tumor cell evasion from the immune system [47]. Metabolic enzymes such as indoleamine-2,3-dioxygenase (IDO) [48–50], which can function to deprive arginine and tryptophan from the microenvironment, are overexpressed in tumor cells, MDSCs, and APCs. Counteracting these critical pathways may be critical in the development of therapeutics for eliciting effective CTL response to tumors.

## 3. Application of Genetically Modified T-Cell-Based Adoptive Immunotherapies: CAR-T and TCR-T-Cell Therapy

**Figure 2:**
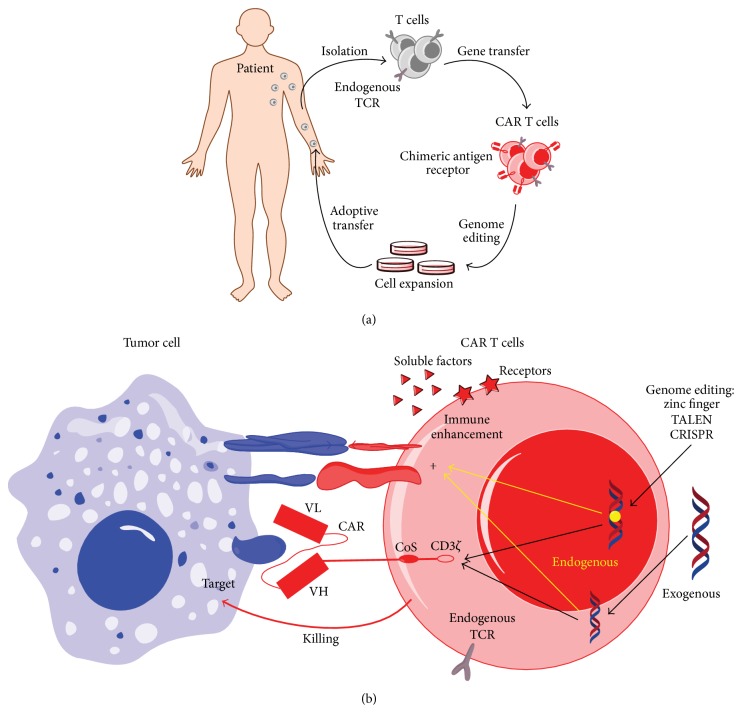
Chimeric antigen receptor (CAR) T-cell-based adoptive immunotherapeutics in hematological malignancies and its targeted recognition on tumor cells in an HLA-independent manner. (a) Flow chart of CAR-T-cell-based adoptive immunotherapeutics. Peripheral blood T-cells isolated from a patient with hematological malignancy are subjected to genetic modification with a relevant CAR that can target the surface antigens of malignant cells. Subsequently, the CAR-modified T-cells are subjected to ex vivo expansion and then administered* via* adoptive transfer to the patient. (b) Tumor cells are recognized and killed by CAR-T-cells in an HLA-independent manner; antitumor immunity can be enhanced and optimized through genome editing and gene transfer technologies.

CTLs are considered to play a key role in antitumor immunity [2], and because impaired recognition of CTLs to tumor cells contributes to immune evasion, regaining the ability of targeted recognition may be a critical component for targeted immunotherapy. Tumor-specific T-cells that are naturally present in patients with malignancies are relatively low, and their function is impaired [14], which combines to contribute to difficulty of T-cell-based adoptive transfer. Currently, the rapid advancement in gene transfer and cell culture technologies has provided a robust basis for redirecting the specificity of CTLs against tumor cells [14]. Genetically modified, patient-derived, T-cells bearing chimeric antigen receptors (i.e., CARs) or neoantigen-specific T-cell receptors (i.e., TCRs) can be generated as therapeutic cellular products with a high level of tumor specificity. The genetically modified T-cells can then be subjected to ex vivo expansion and clinically administered* via* adoptive transfer to patients (Figure 2(a)).

### 3.1. CAR-T-Cell-Based Adoptive Immunotherapy in Hematological Malignancies

CARs are genetically modified receptors (Figure 2(b)) introduced and expressed in human T-cells for targeting the surface antigens of tumor cells in their native conformation [7, 8, 51]. They contain extracellular single-chain variable fragments (ScFv) for antibody-like antigen recognition and intracellular signaling domains for activating T-cells. In CAR-T-cells, the extracellular domain ScFv is responsible for redirecting the specificity of CTLs to the malignant cells and can be designed according to specific antigens such as CD19 expressed in B-cell acute lymphocyte leukemia, chronic lymphocyte leukemia, and lymphoma [7, 52–55]. In contrast, CAR intracellular signaling domains provide the necessary signals for priming T-cell activation [8]. In the CAR-T-cell-mediated immune response, the ScFv of CARs can engage surface antigens of tumors directly* via* antibody-like binding [8]. This occurs in an HLA-independent manner, which is not limited by the presentation of tumor antigens. Thus, specific binding of CARs with surface antigens can facilitate overcoming tumor immune evasion secondary to impaired tumor antigen presentation, thereby promoting the development of personalized CAR-T-cell therapy [8]. In addition, CARs with intracellular costimulatory domains such as CD28 and 4-1BB, which are linked to the CD3*ζ*, can provide additional signals for overcoming immune evasion by priming T-cell activation [1, 2].

CAR-redirected T-cells have exhibited effective response in clinic trails and are considered to be a promising and potential therapy in hematological malignancies [7, 8]. CD19-targeted CAR constructs have been used widely and demonstrate consistently high antitumor activity in patients with relapsed B-cell acute lymphoblastic leukemia, chronic lymphocytic leukemia, and B-cell non-Hodgkin lymphoma. Presently, three generations of CD19-targeted CAR-T-cell-based adoptive immunotherapeutics have been used in clinic trials. The “generations” of CARs typically correlate with the structure of intracellular signaling domains [8]. For the 1st generation, this intracellular signaling domain only contains CD3*ζ* that can transduce an activation signal to the downstream signaling components. Unfortunately, clinic trails indicated that 1st generation of CAR-T-cells resulted in only limited persistence, expansion, and antitumor efficacy [56–58]. Considering that T-cell expansion and persistence require both TCR engagement with peptide-HLA complex and costimulatory signaling, the intracellular domains of the 2nd-generation CARs contain not only CD3*ζ*, but also one costimulatory domain derived from CD28 or 4-1BB. This resulted in dramatic clinical improvement with associated secretion of cytokines and antiapoptotic factors upon antigen engagement [7, 52]. With 3rd-generation CARs the intracellular domains contain two costimulatory domains bearing both CD28 and 4-1BB molecules [7, 8]. However, whether the integration of 4-1BB or/and CD28 into the intracellular domain will ultimately correlate with improved long-term overall survival and event-free survival among patient groups remains to be investigated [7].

Theoretically, CARs can be designed to target and recognize the lineage restricted, nonessential target antigens on the surface of tumor cells in hematological malignancies [59]. Success of CD19-targeted CAR-T-cell therapy is closely related with the nature of CD19: first, CD19 is expressed not only by leukemia cells in patients with B-cell-malignancies, but also by the normal antigen presenting B cells, which can provide additional costimulatory signals for CAR-T-cell activation. Second, depletion of CD19-expressing normal B cells by CD19-targeted CAR-T-cells can produce clinically manageable symptoms [6]. The achievement of CD19-targeted CARs indicates that choosing the optimal target antigens is important for successful CAR-T-cell therapeutics. Recently, cancer-associated Tn glycoform of MUC1, a neoantigen expressed in a variety of cancers, was identified as a promising target that can be recognized by CAR-T-cells. It was observed that anti-Tn-MUC1 CAR-T-cells mediated target-specific cytotoxicity and successfully controlled tumor growth in xenograft models of T-cell leukemia, suggesting the therapeutic efficacy of CAR-T-cells directed against Tn-MUC1. This work identified that aberrantly glycosylated antigens as a novel class of targets can be applied for tumor therapy with engineered T-cells [59, 60]. In ongoing clinic trials, it has been reported that the surface antigens, including CD20, CD30, CD33, CD123, CD38, CD138, Ig *κ* light chain, and Lewis-Y, have been selected as CAR targets in treating leukemia, lymphoma, and multiple myeloma. However, clinical outcome of these approaches remains to be determined [8, 61–68]. Presently, identification of promising targets remains a challenging problem for broadening the use of CAR-T-cell therapy [8].

CAR-related toxicities represent a challenge in the development and popularity of CAR-T-cell therapy. In CD19-targeted CAR-T-cell-based clinic trials, toxicity from CAR-T-cell infusion varied in severity but was similar in clinic manifestation. These toxicities mainly include cytokine release syndrome (CRS) and neurological toxicities [7, 8, 51, 69]. CRS has been described as a systemic inflammatory response syndrome that occurs in the hours to days after CAR-T-cell adoptive transfer, resulting from elevation of proinflammatory cytokines, and T-cell activation and expansion. The clinical features of CRS include fevers, malaise, myalgias, hypoxia, hypotension, renal dysfunction, and coagulopathy [51, 69]. The IL-6 receptor inhibitor tocilizumab as anticytokine therapy or lymphotoxic corticosteroids can be used to treat severe CRS [7]. Clinical reports of neurologic toxicity include headaches, confusion, ataxia, apraxia, facial nerve palsy, alterations in wakefulness, hallucinations, and dysphasia, which are not specific for one area of neuroanatomy [51]. Elevated IL-6 levels and infiltration of anti-CD19 CAR-T-cells in cerebrospinal fluid (CSF) were observed in the patients with neurological toxicities. Tocilizumab and corticosteroids are also candidate drugs for treating neurological toxicities. Vigilant monitoring, aggressive supportive care, early intervention of hypotension, and treatment of concurrent infections are necessary to prevent or treat CAR-related toxicities [51]. Advancement in understanding and management of CAR-related toxicities will promote the overall improvement in the area of CAR-T-cell therapies [7, 8, 51].

### 3.2. Neoantigen Identification and Engineering Neoantigen-Specific TCR-T-Cells

In contrast with CAR-T-cell-based adoptive immunotherapy, which functions by targeting the surface antigens of tumor cells, engineering tumor-reactive TCR-T-cells can instead specifically recognize intracellular tumor antigens presented by HLA molecules [15]. Tumor antigens include tumor-associated antigens, which consist of cancer-testis antigens, tissue differentiation genes, amplified oncogenes, and tumor-specific antigens such as tumor-specific neoantigens [15, 70]. Currently, genes encoding TCRs that are specific for a variety of tumor antigens (including MART-1, gp100, p53, NY-ESO-1, MAGE-A3, and MAGE-A4) have been cloned and used as therapeutic targets for the engineered TCR-T-cell therapy in clinical trials in melanoma, breast cancer, and multiple myeloma [71–77]. This advancement has been reviewed in detail by Hiroaki Ikeda [78].

**Figure 3:**
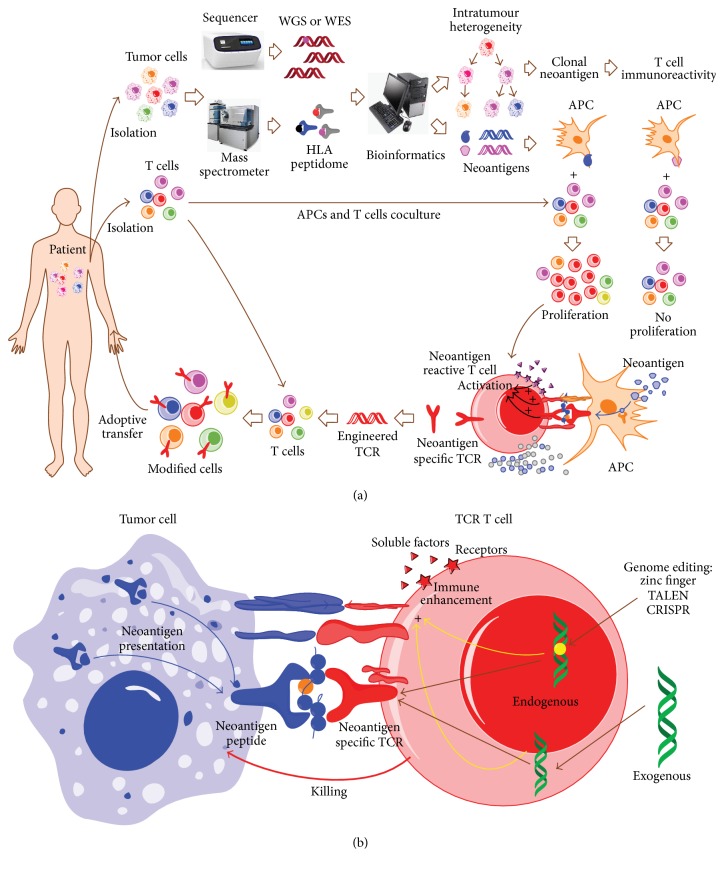
Identification of neoantigens and engineered neoantigen-specific TCR-T-cell-based adoptive immunotherapy in hematological malignancies. (a) Schematic procedures of TCR-T-cell-based adoptive immunotherapy. Both tumor cells and tumor-infiltrating lymphocytes (TIL) are isolated from a patient with hematological malignancies, respectively. The isolated tumor cells are subsequently subjected to gene sequencing (e.g., whole genome sequencing, WGS; whole-exome sequencing, WES), mass spectrometric analysis (e.g., HLA peptidome), and/or bioinformatic analysis, promoting the identification of tumor-specific neoantigens. To validate the immunogenicity of the identified neoantigens, APCs expressing the identified neoantigens are cocultured with the TILs isolated from this patient. The specific population of TILs bearing neoantigen-specific TCRs, which exhibit cell proliferation or cytokine secretion in response to the stimulation of APCs expressing tumor-specific neoantigens, can be isolated, and the neoantigen-specific TCRs can then be cloned successfully. Subsequently, the cloned neoantigen-specific TCRs are transduced into the patient-derived T-cells, generating genetically modified neoantigen-specific T-cells* via* ex vivo activation and expansion. The modified T-cells bearing the neoantigen-specific TCRs can be adoptively transferred to the patient and target tumor cells bearing tumor-specific neoantigens with high specificity for elimination. In addition, the intratumor heterogeneity can be dissected by the single-cell sequencing or other technologies, which can facilitate the identification of clonal neoantigens and thus improve T-cell immunoreactivity. (b) Tumor cells presenting neoantigen derived peptides can be recognized and killed by genetically modified T-cells bearing the responsible neoantigen-specific TCRs. Genome editing and gene transfer technologies and other alternative measures can be utilized to modify the components of other alternative pathways for immune enhancement, ultimately providing an optimized approach to improve TCR-T-cell-based therapeutics.

Neoantigens, derived from the somatic mutations in tumors and representing a unique subset of tumor antigens, play a key role in inhibiting tumor development [15, 79]. They can be identified by next generation sequencing technology and mass spectrometric analysis (Figure 3(a)) [80, 81]. Through the analysis of gene sequencing, it has been demonstrated that each patient with tumor bears a personal mutational profile which translates to a specific clinical manifestation [82, 83]. Additionally, in a given patient, the mutational profile of the tumor in the temporal and spatial dimension dynamically evolves, generating additional layers of intratumor heterogeneity complexity in neoantigen composition [11, 82]. For example, it was recently reported that mutations of tumor cells isolated from the same patient at different sites or at different time are varied [82]. This may facilitate tumor adaptation and therapeutic failure via Darwinian selection. In acute myeloid leukemia, it has been suggested that the malignant founding clone with one or two mutations can yield subclones by acquiring additional cooperating mutations, which can then contribute to disease progression and/or relapse [84]. Neoantigens, which are derived from somatic mutations, exhibit intratumor heterogeneity and can be divided into clonal or subclonal neoantigens [11, 15]. As reported by McGranahan et al. [11], high clonal neoantigen burden and low neoantigen intratumor heterogeneity can lead to prolonged overall survival in primary lung adenocarcinomas. Moreover, CD8^(+)^ tumor-infiltrating lymphocytes reactive to clonal neoantigens can be elicited efficiently and detected in patients with durable clinical benefit (Figure 3(b)). Thus, neoantigen heterogeneity can affect immune surveillance and support the development of therapeutics targeting clonal neoantigens (Figure 3(a)). There are several advantages for clonal neoantigens as therapeutic targets [15]: first, clonal neoantigens are derived from somatic mutations and exclusively expressed by tumor cells [10] and not subject to thymic or peripheral tolerance [10]. This characteristic makes T-cell clones generate higher affinity and specificity to tumors, thereby enhancing immunoreactivity and reducing the potential for off-target toxicity. Second, compared with subclonal neoantigens, clonal neoantigens that can efficiently elicit T-cell immunoreactivity produce an improved clinical benefit [11]. Third, with the rapid development of single-cell sequencing technology, tumor heterogeneity can be described in detail and tumor evolution can be resolved at single-cell level [85], providing the possibility of identifying clonal neoantigens at the individual patient level. The development of single-cell sequencing technology will help design effective and personalized immunotherapeutics for the individual patient, rapidly promoting the development of precision medicine [86]. Thus, identification of neoantigens, especially clonal neoantigens, is critical for personalized immunotherapeutics in the future.

Currently, two main methods to identify neoantigens have been reported. First, computational approaches have been used in epitope prediction [87]. Recently, the mutational profile and* HLA* type of patients with tumors can be identified by next generation sequencing (NGS) in a highly accurate manner, thereby enabling the feasibility of in silico epitope prediction and identification of candidate neoantigens. For example, on the basis of exome sequencing data of chronic lymphocytic leukemia, epitope prediction algorithm NetMHCpan has been used to identify the* HLA*-binding peptides that are derived from leukemia-specific mutations, followed by experimental validation of their binding to HLA-I and quantification of the potential of eliciting a CD8^+^ T-cell response [88]. These similar epitope prediction algorithms have been applied in the identification of other neoantigens [11, 81]. As the mechanisms that determine HLA peptide processing and presentation remain to be fully described, epitope prediction algorithms in silico can yield a large number of false positive hits and identified candidate neoantigens require experimental validation [34]. Second, combining NGS and mass spectrometry analysis may facilitate the process of neoantigen identification. The first example has been reported in melanoma [80]. In parallel to whole-exome analysis on melanoma cells, the HLA class I bound peptides in the same patient's melanoma cells can be purified by the immunoaffinity technology and then subjected to mass spectrometry analysis. Two patient-derived neoantigens that were identified include the P677S alteration in mediator of RNA polymerase II transcription subunit 15 (MED15) and the S123L alteration in Tumor Protein D52-Like 2 (TPD52L2). Experimental validation demonstrated that MED15, but not TPD52L2, elicited the neoantigen-specific T-cell response [34]. Compared with computational approaches [87], this method not only avoided limitations of peptide-MHC binding prediction algorithms in accuracy, but also was less labor-intensive and time-consuming [34]. However, given that somatic mutations have spatial and temporal diversity in individual patients, identifying neoantigens or clonal neoantigens remains a challenging problem.

Only in the past decade have tumor-associated antigens been considered targets for tumor therapy in clinic [9]. The first clinic trial in 2006 in metastatic melanoma demonstrated that adoptive transfer of genetically modified tumor-associated antigen-specific T-cells after host immunodepletion could result in positive clinical outcomes [71]. TCRs that can recognize the tumor-associated antigen MART-1 were transduced into the autologous lymphocytes from peripheral blood of a patient with melanoma, generating engineered tumor-specific T-cells for adoptive immunotherapy [71]. In this clinic trial, durable engraftment at levels exceeding 10% of peripheral blood lymphocytes was observed for at least 2 months after the infusion in 15 patients who received the adoptive transfer. Two patients with high sustained levels of circulating, engineered cells at 1 year after infusion both exhibited regression of metastatic melanoma lesions, suggesting the therapeutic potential of genetically modified T-cells for cancer. Additionally, in a clinic trial of multiple myeloma, which is an incurable hematological malignancy, the adoptive transfer of engineered T-cells that are specific for the cancer-testis antigens NY-ESO-1 and LAGE-1 was well tolerated without clinically apparent CRS, and exhibited an encouraging clinical response. NY-ESO-1-LAGE-1 TCR-engineered T-cells were observed to migrate to marrow and maintain durable persistence that related with clinical activity against antigen-positive myeloma [77]. Compared with certain tumor-associated antigens that are relatively tumor-specific and associated with autoimmunity and tolerance [9], patient-specific neoantigens show greater promise for personalized therapy [10]. Through the application of tumor exome sequencing analysis, a patient-specific neoantigen derived from the mutant epitope of the* ATR* (ataxia telangiectasia and Rad3 related) gene product was identified to elicit a strong T-cell response following ipilimumab treatment. It was also recently reported that tumor-infiltrating neoantigen-reactive CD8^+^ T-cells can be detected in patients with early-stage non-small-cell lung cancer [11]. These studies support the notion that neoantigens that can induce effective antitumor responses in cancer patients may potentially be used as a target in immunotherapy approaches.

The first attempt of adoptive transfer of neoantigen-specific T-cells in clinic trials was reported by Tran et al. in 2014 [12]. This study,* via* a whole-exome sequencing approach, revealed that tumor-infiltrating lymphocytes (TIL) from a patient with metastatic cholangiocarcinoma harbored tumor-derived ERBB2IP (erbb2 interacting protein) mutation-specific CD4^+^ T helper 1 (T_H_1) cells. Following adoptive transfer of TIL containing ERBB2IP mutation-specific polyfunctional T_H_1 cells, a decrease in target lesions was observed with an associated prolonged stabilization of disease. These observations provide evidence that a CD4^+^ T-cell response against a neoantigen derived from a patient-specific mutation could be used to promote regression of a metastatic epithelial cancer. Recently, in two patients with stage IV melanoma the dynamic interaction of neoantigen-specific T-cell responses with their recognition antigens treated by adoptive T-cell transfer was observed [89]. Likely due to overall reduced expression of the genes or loss of the mutant alleles, the T-cell-recognized neoantigens were selectively lost, which was accompanied by development of neoantigen-specific T-cell reactivity in tumor-infiltrating lymphocytes. This work suggests that T-cells have intrinsic capacity to contribute to neoantigen immunoediting and broad neoantigen-specific T-cell responses and could be used to avoid tumor resistance in the future [89]. Recognition of neoantigens may serve as a major driving force behind the approaches incorporating immunotherapy with adoptive T-cell and T-cell checkpoint blockade [81, 90–95], which may ultimately support the development of strategies to selectively elicit T-cell reactivity, advancing the field of personalized therapies for hematologic malignancies.

## 4. Optimization of Genetically Modified T-Cell-Based Adoptive Immunotherapy

By enhancing the targeted recognition of tumor-specific antigens, genetically modified T-cell based adoptive immunotherapeutics, including CAR-T and TCR-T-cell therapies, can promote recognition and targeting of tumor cells in an HLA-independent or HLA-dependent manner, thereby promoting the elimination of tumor cells. However, there remain significant obstacles limiting T-cell-based adoptive immunotherapeutics in efficacy and toxicity. Therefore, optimal measures promoting immune enhancement and/or reduced toxicity in approaches that utilize genetically modified T-cell-based adoptive immunotherapy should consider the following: first, in order to enhance efficacy of the T-cell response, the combination of genetically modified T-cell-based adoptive immunotherapy with other measures that interfere with the pathways or steps for tumor immune evasion should be considered. As discussed above, tumor cells utilize multiple mechanisms to escape immunity-mediated elimination [1, 2]. Combinational therapies that simultaneously target multiple pathways for immune evasion can be applied to improve the likelihood of an effective clinical outcome. For example, lymphodepletion chemotherapy, which can eliminate suppressive immune cells that release inhibitory soluble factors or directly block the CTL response and promote tumor antigen presentation [96, 97], may enhance the antitumor efficacy of CAR-T-cell therapy [7]. Inadequate lymphodepletion chemotherapy was considered a factor contributing to limited CAR-T-cell persistence and reduced clinic efficacy [98]. Further, therapeutic blockade of immune checkpoints by blocking antibodies against CTLA-4 or PD-1 has been shown to produce a broad and beneficial clinical outcome in hematological malignancies [20], as well as improving the potency of CAR-T-cell or TCR-T-cell-based therapies [11, 81, 99, 100]. A recent report showed that PD-1/PD-1 ligand [PD-L1] pathway interference through PD-1 antibody checkpoint blockade reactivated the effector function of exhausted CD28 CAR-T-cells and enhanced efficacy of CAR-T therapy in an orthotopic mouse model of pleural mesothelioma [100]. In addition, neoantigens serve as a major class of T-cell rejection antigens following anti-PD-1 and/or anti-CTLA-4 therapy, supporting approaches that utilize a combination of different checkpoint blockade treatments with neoantigen-specific T-cell therapies [81].

A second consideration for approaches that utilize genetically modified T-cell-based adoptive immunotherapy is with the incorporation of adjunct methodologies such as genome editing and gene transfer technologies, in order to enhance efficacy and reduce toxicity. It has been reported that gene transfer and genome editing technologies provide a feasible platform for using genetic engineering to add or remove genes in therapeutic T-cells [14, 101]. For example, the inhibition of mammalian target of rapamycin complex 1 (mTORC1) signaling by immune-suppressive cytokines such as transforming growth factor *β* impairs T-cell activation. Ras homolog overexpressed in engineered T-cells was shown to contribute to the upregulation of mTORC1 signaling, which has improved eradication of established tumors following adoptive T-cell therapy [102]. Genome editing technologies (including ZFN, TALEN, and CRISPR) has been reported to optimize genetically engineered T-cell therapy [14]. Given that exogenous TCR can mismatch and compete with endogenous TCRs in engineered T-cells, gene transfer approaches may produce suboptimal activity and potentially harmful unpredicted antigen-specific targeting. Recently, lymphocytes treated with ZFNs, which were designed to promote the disruption of endogenous TCR and chain genes, were transduced with a TCR specific for the Wilms tumor 1 antigen. These endogenous TCR-edited cells expressed high levels of the transduced exogenous TCR gene and did not produce off-target reactivity, while maintaining their antitumor activity in vivo, thereby demonstrating that genome editing technology could be potentially applied to optimizing engineered T-cell therapy [103]. Moreover, it was demonstrated that the replacement of key residues in the framework of the variable region in engineered TCRs could result in their high affinity and expression, thereby enhancing their therapeutic potency [104].

## 5. Conclusions

Only with a comprehensive understanding of the multiple mechanisms of tumor immune evasion can the development of genetically modified T-cell-based adoptive immunotherapeutics promise to treat or cure patients with hematologic tumors being realized. Clinic trials of CAR-T or TCR-T-cell therapy in hematological malignancies and other solid tumors such as melanoma provide a series of successful examples to validate the efficacy and safety of this approach in the clinic. In future approaches, three points should be carefully considered: (1) identification of new targets, including tumor-specific surface molecules and neoantigens, and utilizing and integration of* omics* science with immunology; (2) a detailed understanding of the cooperation and interaction of T-cell-based adoptive immunotherapies with other treatments in the design of an optimal combinational therapy; and (3) application of optimal measures incorporating genome editing and gene transfer technologies, to enhance efficacy and reduce toxicity, facilitating future development and clinical incorporation of this rapidly advancing technology.
